# Development and Validation of a Clinical‐Ultrasonographic Model for Differentiating Follicular Thyroid Carcinoma From Adenoma in a Surgically Selected Cohort

**DOI:** 10.1155/ije/2627742

**Published:** 2026-11-14

**Authors:** Minghui Ren, Mengshu Lu, Yajie Li, Dai Cui

**Affiliations:** ^1^ Department of Endocrinology The First Affiliated Hospital with Nanjing Medical University Nanjing Jiangsu China njmu.edu.cn; ^2^ Department of General Practice The First Affiliated Hospital with Nanjing Medical University Nanjing Jiangsu China njmu.edu.cn

**Keywords:** follicular adenoma, follicular thyroid carcinoma, predictive model, ultrasound

## Abstract

**Purpose:**

To identify clinical and ultrasonographic features that distinguish follicular thyroid carcinoma (FTC) from follicular adenoma (FA) and to develop and validate a predictive model for the preoperative risk stratification of follicular neoplasms (FNs).

**Methods:**

In this retrospective study, patients who underwent thyroid surgery due to FNs were consecutively enrolled. The training cohort comprised 142 patients with FA and 48 with FTC diagnosed between January 2016 and December 2022. A temporal validation cohort included 41 FA and 21 FTC cases diagnosed between January 2023 and August 2024. Demographic characteristics, ultrasound features, and serological markers were analyzed using univariate analysis and multivariate logistic regression to identify independent predictors of FTC. A predictive nomogram was constructed based on the identified risk factors. Model performance was evaluated in both cohorts using receiver operating characteristic (ROC) analysis, calibration plots, and decision curve analysis (DCA) to assess discrimination, calibration, and potential clinical utility, respectively. Given the potential interference of thyroglobulin antibody (TgAb) with serum thyroglobulin (Tg) measurements, a subgroup analysis was performed among TgAb‐negative patients. This subgroup included 122 FA and 53 FTC patients diagnosed between January 2016 and August 2024.

**Results:**

In the predictive model, age (per 10‐year increase; odds ratio [OR]: 0.652, *p* = 0.002), elevated log_2_‐transformed serum Tg (OR: 1.271, *p* = 0.030), larger maximum diameter (per 10‐mm increase; OR: 1.744, *p* < 0.001), hypoechogenicity (OR: 3.188, *p* = 0.008), and irregularity (OR: 12.295, *p* < 0.001) were independently associated with FTC. The resulting model showed good discrimination, with areas under the curve (AUCs) of 0.822 (95% confidence interval [CI]: 0.753–0.891) in the training cohort, 0.801 (95% CI: 0.686–0.917) in the temporal validation cohort, and 0.814 (95% CI: 0.745–0.882) in the TgAb‐negative sensitivity analysis. Calibration analyses showed generally acceptable agreement between predicted and observed probabilities, although estimates in the temporal validation cohort were imprecise. DCA suggested potential net clinical benefit across a range of threshold probabilities.

**Conclusion:**

In this surgically selected cohort, the clinical‐ultrasonographic model showed potentially useful discrimination between FTC and FA. The model should be regarded as an exploratory risk stratification tool for patients similar to those included in this study. Its applicability to unselected patients with cytologically indeterminate thyroid nodules and its ability to inform decisions for or against diagnostic surgery require prospective external validation.

## 1. Introduction

Follicular neoplasms (FNs) of the thyroid gland originate from thyroid follicular epithelium and mainly comprise benign follicular adenoma (FA) and malignant follicular thyroid carcinoma (FTC) [1]. FA accounts for approximately 60%–75% of FNs, whereas FTC represents 10%–40% [2]. FTC constitutes about 10%–15% of all thyroid carcinomas and is the second most common histological subtype after papillary thyroid carcinoma (PTC) [3]. Unlike PTC, which predominantly spreads via the lymphatic system, FTC shows a strong propensity for hematogenous dissemination. Distant metastases occur in approximately 15%–27% of FTC cases, most commonly involving the bone and lungs [4]. Patients presenting with distant metastases exhibit significantly lower survival rates, with 10‐year overall survival reported as only 23%–28% [5]. Therefore, early identification and timely surgical intervention are critical for improving outcomes in FTC. However, reliable preoperative differentiation between FTC and FA remains challenging because of overlapping ultrasonographic features, the absence of specific serum or tumor biomarkers, and the limited diagnostic sensitivity of fine‐needle aspiration cytology (FNAC) [6].

Ultrasonography (US) is the first‐line imaging modality for evaluating thyroid nodules. Nonetheless, most ultrasonographic features traditionally associated with malignancy—such as hypoechogenicity, irregular margins, microcalcifications, and a taller‐than‐wide shape—are derived primarily from studies focusing on PTC [7]. While these features demonstrate high diagnostic accuracy for PTC, their sensitivity in distinguishing FTC from FA is relatively low. Previous studies investigating the ultrasonographic characteristics of FTC have reported inconsistent results, largely owing to heterogeneity in study design, sample size, and patient populations [8–10].

Thyroglobulin (Tg), synthesized by thyroid follicular epithelial cells, is widely recognized as a pivotal serological marker for the diagnosis and postoperative surveillance of differentiated thyroid carcinoma (DTC) [11]. Li et al. [12] reported that elevated preoperative serum Tg levels are associated with an increased risk of FTC and may facilitate differentiation between FA and FTC before surgery. The thyroglobulin antibody (TgAb) and thyroid peroxidase antibody (TPOAb) are crucial parameters reflecting the autoimmune status of the thyroid [13]. Significant associations between thyroid autoimmune status and thyroid cancer development have been reported in prior investigations [14–16]. Chen et al. [17] demonstrated that preoperative elevation of serum TgAb levels represented an independent predictor for FTC, while no significant correlation was observed between TPOAb levels and FTC incidence. However, to date, investigations into the correlation between serum thyroid hormone profiles and FTC have remained limited, with the nature of their relationship remaining inconclusive and necessitating further exploration.

FNAC is widely regarded as the standard preoperative diagnostic approach for thyroid nodules. However, the definitive diagnosis of FTC relies on histopathological evidence of capsular and/or vascular invasion [18]. Because these features cannot be evaluated using FNAC, its diagnostic utility in distinguishing FTC from FA is inherently limited [19]. Notably, only 23%–34% of nodules classified as Bethesda Category IV are ultimately malignant [20], indicating that most patients undergoing diagnostic surgery have benign disease and highlighting the suboptimal performance of FNAC in this setting.

In cases of indeterminate cytological findings in thyroid nodules, additional molecular testing is recommended. RAS mutations are the most frequently observed genetic alterations in FTC, occurring in approximately 40%–50% of cases, with NRAS mutations being the most prevalent. However, RAS mutations are also detected in 20%–30% of FAs [21]. Other less common but significant genetic alterations in FTC include PPARγ rearrangements, EIF1AX, DICER1, and PTEN mutations, which have also been identified in a subset of FAs [22, 23]. In contrast to the diagnostic specificity of the BRAF^*V600E*^ mutation for PTC [24], no reliable single‐gene assay is currently available to differentiate benign from malignant FN, and multigene panels may only partially improve diagnostic accuracy.

Given the limited diagnostic value of any single preoperative assessment, there is growing interest in multifactorial predictive models integrating diverse parameters. Accordingly, this study aimed to identify risk factors associated with FTC and to develop a predictive model based on comprehensive preoperative clinical data, including demographic characteristics, thyroid serological markers, and ultrasonographic features, in order to facilitate exploratory preoperative risk stratification for differentiating FTC from FA in surgical patients.

## 2. Materials and Methods

### 2.1. Patients

This was a single‐center retrospective prediction model development and temporal validation study. The study flow diagram is illustrated in Figure 1. A total of 252 patients who underwent thyroid surgery at the First Affiliated Hospital with Nanjing Medical University and were histopathologically diagnosed with FTC (*n* = 69) or FA (*n* = 183) between January 2016 and August 2024 were included. Patients diagnosed between January 2016 and December 2022 (FTC = 48, FA = 142) were allocated to the training cohort, whereas those diagnosed between January 2023 and August 2024 (FTC = 21, FA = 41) comprised the temporal validation cohort. Given that serum Tg levels may be influenced by TgAb, a subgroup analysis, not an independent validation, was performed in TgAb‐negative patients (TgAb ≤ 115.00 IU/mL), including 53 FTC and 122 FA cases treated between January 2016 and August 2024. The study protocol was approved by the Ethics Committee of the First Affiliated Hospital with Nanjing Medical University (Ethics No. 2024‐SR‐455), and the requirement for written informed consent was waived by the Ethics Committee owing to the retrospective study design.

**FIGURE 1:**
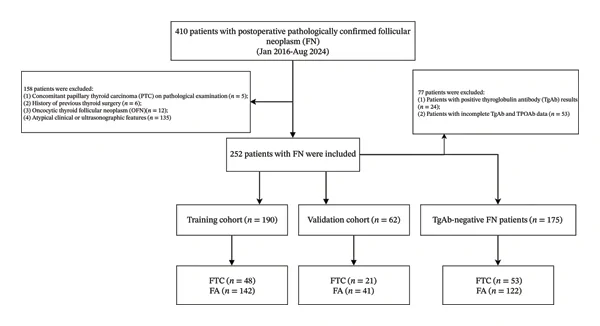
Flow diagram of patient selection and cohort allocation. FTC, follicular thyroid carcinoma; FA, follicular adenoma.

The intended‐use population of the present model was restricted to patients with follicular thyroid neoplasms who underwent surgical resection and subsequently received a definitive histopathological diagnosis of FTC or FA. Because eligibility required surgery and histopathological confirmation, nonoperated nodules and patients managed with active surveillance were not represented. Therefore, the present study was not designed to determine whether the model could safely rule out malignancy or obviate diagnostic surgery in an unselected population of patients with cytologically indeterminate thyroid nodules.

Inclusion criteria were as follows: (1) postoperative histopathological confirmation of FA or FTC and (2) availability of complete clinical and ultrasonographic data. Exclusion criteria included (1) the presence of thyroid tumor subtypes other than FTC or FA, (2) prior thyroid surgery, and (3) concomitant malignancies in other organ systems.

### 2.2. Clinical Characteristics

Demographic and preoperative thyroid serological data, including age, sex, and laboratory markers, were collected. Preoperative thyroid serological parameters were measured in the central laboratory of our institution. Serum Tg was quantified using the Elecsys Tg II electrochemiluminescence immunoassay (Roche Diagnostics GmbH, Mannheim, Germany) on a MODULAR ANALYTICS E170 analyzer. The assay uses a two‐step double‐antibody sandwich principle and has an analytical measuring range of 0.04–500 ng/mL. Calibration was traceable to the certified reference material BCR‐CRM 457. The reference interval for Tg was 3.50–77.00 ng/mL. Serum FT3, FT4, TSH, TgAb, and TPOAb were measured using the same electrochemiluminescence platform and matching Roche reagent kits. The corresponding reference intervals were 3.10–6.80 pmol/L, 12.00–22.00 pmol/L, 0.270–4.200 mIU/L, ≤ 115.00 IU/mL, and ≤ 34.00 IU/mL, respectively. Samples exceeding the analytical measuring range were not routinely subjected to dilution testing; therefore, their exact Tg concentrations above 500 ng/mL were unavailable. Results reported as > 500 ng/mL were recorded as 500 ng/mL in the study database and were considered values recorded at the upper reporting limit. Similarly, the lower limit of detection for TSH was established as 0.01 mIU/L, and values below this level were assigned a value of 0.01 mIU/L. The upper reporting limit for TPOAb was 600 IU/mL, and values exceeding this threshold were recorded as 600 IU/mL.

### 2.3. Ultrasonographic Characteristics

Ultrasonographic features assessed for each nodule included diameter, composition (solid, cystic, or mixed), echogenicity (hypoechogenicity, isoechogenicity, hyperechogenicity, or mixed echogenicity), calcification (absent, microcalcification, macrocalcification, or peripheral calcification), taller than wide (absent or present), location (left lobe, right lobe, or isthmus), regularity (regular or irregular), margin (smooth or irregular), peripheral halo (present or absent), and blood supply (rich or scarce). All ultrasound examinations were performed by experienced sonographers with at least 5 years of clinical experience who had completed standardized training and competency assessments.

### 2.4. Inter‐reader Reproducibility Assessment

To evaluate the reproducibility of the ultrasonographic features, a stratified random sample of 120 patients with complete and diagnostically adequate archived ultrasound images was included in the reproducibility assessment. The images were independently rereviewed by two sonographers with 10 and 12 years of experience in thyroid US, respectively. Both readers were blinded to the final histopathological diagnosis, laboratory findings, original ultrasound reports, original feature coding, and each other’s assessments. Before image review, the readers agreed on standardized definitions for all evaluated features. Shape was classified as regular when the nodule was round or oval with a generally symmetric contour and as irregular when it was nonround/nonoval, asymmetric, or lobulated. Hypoechogenicity was defined as echogenicity lower than that of the adjacent normal thyroid parenchyma. Inter‐reader agreement was assessed using the independent ratings obtained before any consensus discussion.

### 2.5. Statistical Analysis

Statistical analyses were conducted using MedCalc 22.0, SPSS 27.0, and R 4.5.3. Qualitative data were reported as frequency and percentage (%), with comparisons between the FTC and FA groups performed using the chi‐square test or Fisher’s exact test. Quantitative data were expressed as mean ± SD or median (25th–75th percentile), with group comparisons performed via the *t*‐test or Mann–Whitney *U* test. Restricted cubic splines (RCSs) with three knots, located at the 10th, 50th, and 90th percentiles of the distribution of each continuous variable, were employed to flexibly model and visualize potential nonlinear relationships between continuous variables and the risk of FTC. Variables significant in univariate analysis were included in multivariable logistic regression to identify independent predictors of FTC. The stability of variable selection was assessed using bootstrap resampling (*B* = 2000), in which a logistic regression model was fitted in each iteration and variables with *p* < 0.05 (ANOVA likelihood ratio test) were considered selected. The bootstrap inclusion frequency was calculated as the proportion of the 2000 resamples in which each variable was retained. Model performance was evaluated using receiver operating characteristic (ROC) curves, calibration plots, bootstrap‐derived calibration slope and calibration intercept, and decision curve analysis (DCA). The optimal threshold was identified by maximizing the Youden index. Sensitivity, specificity, positive and negative predictive values (PPV, NPV), and positive and negative likelihood ratios (PLR, NLR) were calculated with 95% confidence intervals (CIs). For sensitivity, specificity, PPV, and NPV, 95% CIs were calculated using the Wilson score method; likelihood ratio CIs were calculated on the log scale. Because PPV and NPV depend on disease prevalence, prevalence‐standardized predictive values were additionally calculated for prespecified pretest probabilities of 10%, 15%, 20%, 25%, and 30% using Bayes’ theorem while holding the sensitivity and specificity observed in the temporal validation cohort fixed. Uncertainty in the standardized PPV and NPV estimates was quantified using 2000 stratified bootstrap resamples of the temporal validation cohort. In addition, an exploratory rule‐out‐oriented operating point was derived exclusively in the training cohort as the highest predicted‐probability threshold achieving a sensitivity of at least 90%. This threshold was then applied without modification to the temporal validation cohort. Pairwise area under the curve (AUC) comparisons were assessed using the paired DeLong test. Incremental predictive value was further evaluated by continuous net reclassification improvement (NRI) and integrated discrimination improvement (IDI), with 95% CIs derived from 2000 stratified bootstrap resamples.

For benchmarking against an established ultrasonographic risk stratification system, an ACR TI‐RADS point score was retrospectively reconstructed for each nodule from the recorded ultrasonographic descriptors according to the ACR TI‐RADS framework [25]. Composition was scored as 2 points for solid nodules and 1 point for mixed cystic–solid nodules; hyperechogenicity/isoechogenicity and hypoechogenicity were assigned 1 and 2 points, respectively; a taller‐than‐wide shape was assigned 3 points; an irregular margin was assigned 2 points; and macrocalcification, peripheral/rim calcification, and punctate echogenic foci were assigned 1, 2, and 3 points, respectively. Three nodules recorded as having mixed echogenicity were assigned 1 point using the ACR recommendation when echogenicity cannot be determined. Sensitivity analyses assigning these three nodules 2 or 3 points did not materially alter discrimination. Because the retrospective database recorded a single calcification/echogenic foci category for each nodule, the reconstructed score reflects the recorded focus type and could not account for potentially coexisting unrecorded echogenic foci. An ACR TI‐RADS comparator model using the total point score as a continuous predictor was fitted in the training cohort and applied without modification to the temporal validation cohort. Its discrimination, incremental performance, and clinical net benefit relative to the combined model were evaluated using AUC, paired DeLong testing, continuous NRI, IDI, and DCA.

The training cohort contained 48 FTC events. Relative to the six candidate predictors considered during model development, the events‐per‐variable ratio was 8.0; relative to the five predictors retained in the final model, it was 9.6. Because the limited event count, data‐driven variable selection, and sparse distribution of irregularity could increase the risk of small‐sample and sparse‐data bias, a sensitivity analysis was performed using Firth penalized logistic regression (logistf package in R). The Firth model included the same five predictors as the primary multivariable model—age (per 10‐year increase), log_2_‐transformed Tg, maximum nodule diameter (per 10‐mm increase), hypoechogenicity, and irregularity—without further variable selection. Penalized maximum‐likelihood estimates with profile penalized‐likelihood 95% CIs were compared with the conventional maximum‐likelihood estimates.

Continuous predictors were modeled in their continuous form. Serum Tg was log_2_‐transformed to address its marked right skewness, with the odds ratio interpreted per doubling; age and maximum nodule diameter were scaled per 10‐year and 10‐mm increases, respectively. Serum Tg values exceeding the analytical measuring range were recorded as 500 ng/mL, and the proportions of patients with Tg values at this upper reporting limit were reported separately by diagnosis and cohort. To assess the potential influence of upper‐limit censoring, two sensitivity analyses were performed. First, an indicator variable denoting a Tg value at the upper reporting limit was added to the primary multivariable model. Second, the same model was refitted after excluding patients whose Tg values were recorded at 500 ng/mL. The direction and magnitude of the Tg coefficient and model discrimination were compared with those of the primary analysis.

Inter‐reader agreement for binary ultrasonographic features was assessed using Cohen’s *κ* with 95% CIs. Observed agreement and positive and negative agreement were additionally reported because *κ* may be influenced by feature prevalence.

## 3. Results

### 3.1. Clinical and Ultrasonographic Characteristics

A total of 252 patients with histopathologically confirmed FTC (*n* = 69) or FA (*n* = 183) treated between January 2016 and August 2024 were enrolled. Patients were allocated to a training cohort (FTC = 48, FA = 142; January 2016–December 2022) and a validation cohort (FTC = 21, FA = 41; January 2023–August 2024) using a temporal split. Baseline demographic characteristics, thyroid serological parameters, and ultrasonographic features of patients in the training cohort are summarized in Table 1. No significant difference in sex distribution was observed between the FTC and FA groups. However, patients with FNs who were younger at presentation exhibited a higher risk of malignancy. The median age was significantly lower in the FTC group than in the FA group (39.00 years [IQR: 27.50–53.75] vs. 49.00 years [IQR: 39.00–60.00], *p* = 0.004). The median preoperative serum Tg level was significantly higher in the FTC group compared with the FA group (185.15 ng/mL [IQR: 63.27–500.00] *vs.* 60.12 ng/mL [IQR: 19.75–207.70], *p* < 0.001). No statistically significant differences were observed in preoperative serum FT3, FT4, or TSH levels between the two groups (*p* = 0.280, *p* = 0.371, *p* = 0.835, respectively). Due to missing TgAb and TPOAb data in 8 FTC patients and 45 FA cases, a comparative analysis was not performed. Compared with FA, FTC lesions were more frequently characterized by larger maximum diameter (*p* < 0.001), hypoechogenicity (*p* = 0.012), presence of calcification (*p* = 0.031), and irregular shape (*p* < 0.001). No statistically significant differences were observed between the two groups with respect to the number of nodules, lesion location, composition, margin characteristics, taller‐than‐wide shape, peripheral halo, or blood supply (all *p* > 0.05). Consistent with the surgically selected nature of the cohort, the included nodules were relatively large. In the training cohort, the median maximum diameters were 46.20 mm in patients with FTC and 36.00 mm in patients with FA.

**TABLE 1:** Clinical and ultrasonographic characteristics of patients in the training cohort.

	FTC (*n* = 48)	FA (*n* = 142)	*p* value
Gender, *n* (%)			0.124
Male	13 (27.10%)	24 (16.90%)	
Female	35 (72.90%)	118 (83.10%)	
Age (year)	39.00 (27.50–53.75)	49.00 (39.00–60.00)	0.004^∗^
FT3 (pmol/L)	4.71 (4.28–5.12)	4.86 (4.46–5.21)	0.280
FT4 (pmol/L)	15.79 (14.10–17.56)	16.10 (14.50–17.57)	0.371
TSH (mIU/L)	1.81 (0.98–3.25)	1.91 (1.30–3.09)	0.835
Tg (ng/mL)	185.15 (63.27–500.00)	60.12 (19.75–207.70)	< 0.001^∗^
Maximum diameter (mm)	46.20 (38.25–55.13)	36.00 (25.75–46.25)	< 0.001^∗^
Number, *n* (%)			0.415
Single	19 (39.60%)	47 (33.10%)	
Multiple	29 (60.40%)	95 (66.90%)	
Location, *n* (%)			1.000
Left	21 (43.75%)	61 (43.00%)	
Right	27 (56.25%)	80 (56.30%)	
Isthmus	0	1 (0.70%)	
Composition, *n* (%)			0.066
Solid	32 (66.70%)	73 (51.40%)	
Cystic or mixed	16 (33.30%)	69 (48.60%)	
Echogenicity, *n* (%)			
Type 1			0.045^∗^
Isoechogenicity	15 (31.25%)	62 (43.70%)	
Hypoechogenicity	33 (68.75%)	68 (47.90%)	
Hyperechogenicity	0	9 (6.30%)	
Mixed echogenicity	0	3 (2.10%)	
Type 2			
Hypoechogenicity, *n* (%)			0.012^∗^
Present	33 (68.75%)	68 (47.90%)	
Absent	15 (31.25%)	74 (52.10%)	
Calcification, *n* (%)			
Type 1			0.128
Absent	23 (47.90%)	93 (65.50%)	
Microcalcification	14 (29.20%)	30 (21.10%)	
Macrocalcification	10 (20.80%)	16 (11.30%)	
Peripheral calcification	1 (2.10%)	3 (2.10%)	
Type 2			0.031^∗^
Present	25 (52.10%)	49 (34.50%)	
Absent	23 (47.90%)	93 (65.50%)	
Regularity, *n* (%)			< 0.001^∗^
Regular	37 (77.10%)	135 (95.10%)	
Irregular	11 (22.90%)	7 (4.90%)	
Margin, *n* (%)			0.262
Smooth	40 (83.30%)	127 (89.40%)	
Irregular	8 (16.70%)	15 (10.60%)	
Taller than wide, *n* (%)			0.988
Present	1 (2.10%)	5 (3.50%)	
Absent	47 (97.90%)	137 (96.50%)	
Peripheral halo, *n* (%)			0.517
Present	9 (18.75%)	33 (23.20%)	
Absent	39 (81.25%)	109 (76.80%)	
Blood supply, *n* (%)			0.059
Rich	41 (85.40%)	102 (71.80%)	
Scarce	7 (14.60%)	40 (28.20%)	

*Note:* FT3, free triiodothyronine; FT4, free thyroxine; Tg, thyroglobulin.

Abbreviations: FA, follicular adenoma; FTC, follicular thyroid carcinoma; TSH, thyroid‐stimulating hormone.

^∗^Statistically significant difference.

Serum Tg values were recorded at the upper reporting limit of 500 ng/mL in 21 of 69 patients with FTC (30.40%) and 18 of 183 patients with FA (9.80%) in the overall cohort (Fisher’s exact *p* < 0.001). The corresponding proportions were 31.30% versus 9.90% in the training cohort and 28.60% versus 9.80% in the temporal validation cohort, respectively (Table 2).

**TABLE 2:** Distribution of serum thyroglobulin values recorded at the upper reporting limit according to diagnosis and cohort.

**Cohort**	**Diagnosis**	**Total *N***	**Tg at 500 ng/mL, *n* (%)**	**OR (95% CI)**	**p**

*Panel A. Distribution of serum Tg values recorded at the upper reporting limit*					
Overall	FA	183	18 (9.80)	Reference	
Overall	FTC	69	21 (30.40)	3.984 (1.856–8.652)	< 0.001
Training	FA	142	14 (9.90)	Reference	
Training	FTC	48	15 (31.30)	4.117 (1.671–10.257)	< 0.001
Temporal validation	FA	41	4 (9.80)	Reference	
Temporal validation	FTC	21	6 (28.60)	3.613 (0.737–20.067)	0.075

**Analysis**	**Tg OR per doubling (95% CI)**	**p**	**Training AUC (95% CI)**	**Temporal validation AUC (95% CI)**	

*Panel B. Sensitivity analyses addressing values recorded at the upper reporting limit*					
Primary model	1.271 (1.024–1.579)	0.030	0.822 (0.753–0.891)	0.801 (0.686–0.917)	
Model plus upper‐limit indicator	1.232 (0.946–1.605)	0.121	0.826 (0.757–0.894)	0.794 (0.676–0.913)	
Exclusion of upper‐limit values	1.224 (0.925–1.619)	0.157	0.823 (0.738–0.907)	0.831 (0.692–0.969)	

*Note:* Tg, thyroglobulin.

Abbreviations: AUC, area under the curve; CI, confidence interval; OR, odds ratio.

The RCS analysis revealed that age (per 10‐year increase) and maximum tumor diameter (per 10‐mm increase) exhibited significant linear associations with FTC risk, with no evidence of deviation from linearity (both *p* for nonlinearity > 0.05). Specifically, the odds of FTC decreased linearly with advancing age (*p* overall = 0.005) (Figure 2a), whereas larger tumor diameter was significantly associated with an increased risk of FTC (*p* overall = 0.001) (Figure 2b). In contrast, log_2_ (Tg) did not demonstrate a statistically significant nonlinear or overall association with FTC risk in the RCS analysis (*p* nonlinear = 0.716, *p* overall = 0.096) (Figure 2c).

**FIGURE 2:**
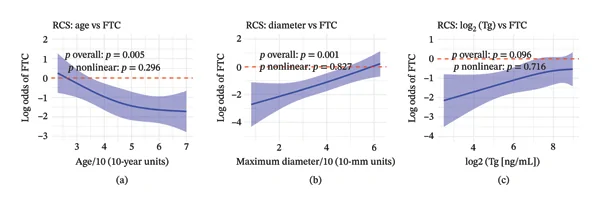
RCS analysis of the association between age (per 10‐year increase), log_2_ (Tg), maximum diameter (per 10‐mm increase), and the risk of FTC. (a) The RCS term was fitted in a multivariable logistic regression model adjusted for log_2_ (Tg), maximum diameter (per 10‐mm increase), hypoechogenicity, and irregularity. (b) The RCS term was fitted in a multivariable logistic regression model adjusted for age (per 10‐year increase), log_2_ (Tg), hypoechogenicity, and irregularity. (c) The RCS term was fitted in a multivariable logistic regression model adjusted for age (per 10‐year increase), maximum diameter (per 10‐mm increase), hypoechogenicity, and irregularity. RCS, restricted cubic spline; FTC, follicular thyroid carcinoma; Tg, thyroglobulin.

### 3.2. Inter‐reader Reproducibility of Ultrasonographic Features

A total of 120 patients, including 33 patients with FTC and 87 with FA, were included in the inter‐reader reproducibility assessment. The two readers agreed on the classification of irregularity in 96.70% of cases, with a Cohen’s *κ* of 0.880 (95% CI: 0.765–0.995). For hypoechogenicity, the observed agreement was 95.80%, and Cohen’s *κ* was 0.914 (95% CI: 0.839–0.988). These results indicated almost perfect inter‐reader agreement for both ultrasonographic features. Full inter‐reader agreement results are presented in Table 3.

**TABLE 3:** Inter‐reader reproducibility of ultrasonographic features.

Feature	Reader 1 positive, *n* (%)	Reader 2 positive, *n* (%)	Observed agreement (%)	Cohen’s *κ*	95% CI	Positive agreement (%)	Negative agreement (%)
Irregularity	20 (16.70)	20 (16.70)	96.70	0.880	0.765–0.995	90.00	98.00
Hypoechogenicity	73 (60.80)	70 (58.30)	95.80	0.914	0.839–0.988	96.50	94.80

Abbreviation: CI, confidence interval.

### 3.3. Development of the Clinical and Ultrasonographic Diagnostic Model

Based on clinical relevance and univariate analysis (*p* < 0.05), six variables—age, Tg, maximum diameter, hypoechogenicity, regularity, and calcification—were initially entered into the multivariable logistic regression. To address the marked right‐skewed distribution of serum Tg, a log_2_‐transformation was applied, with the resulting odds ratio interpreted per doubling of concentration. Age and maximum nodule diameter were modeled per 10‐year and 10‐mm increments, respectively. Internal validation was performed using the bootstrap method with 2000 resamples to assess model stability. Inclusion frequencies varied among predictors: Maximum diameter (98.30%) and irregularity (90.80%) demonstrated high robustness (> 90%), followed by age (86.65%) and hypoechogenicity (71.95%). Tg demonstrated a moderate bootstrap inclusion frequency (54.50%), indicating lower selection stability relative to other retained predictors. It was retained in the final model owing to its well‐established clinical significance; however, its contribution was interpreted with caution and further evaluated through sensitivity analyses. Conversely, calcification was excluded from the final model, as its inclusion frequency (34.60%) fell below the prespecified stability threshold.

Multivariate binary logistic regression analysis identified age (per 10‐year increase, OR = 0.652, *p* = 0.002), elevated log_2_‐transformed serum Tg (OR = 1.271, *p* = 0.030), larger maximum diameter (per 10‐mm increase, OR = 1.744, *p* < 0.001), hypoechogenicity (OR = 3.188, *p* = 0.008), and irregularity (OR = 12.295, *p* < 0.001) as independent predictors of FTC (Table 4).

**TABLE 4:** Multivariate logistic regression analysis in the training cohort.

Variable	*β*	SE	OR (95% CI)	*p*
Age (per 10‐year increase)	−0.428	0.140	0.652 (0.495–0.857)	0.002
Log_2_ (Tg)	0.240	0.111	1.271 (1.024–1.579)	0.030
Diameter (per 10‐mm increase)	0.556	0.147	1.744 (1.308–2.326)	< 0.001
Hypoechogenicity	1.159	0.436	3.188 (1.357–7.486)	0.008
Irregularity	2.509	0.702	12.295 (3.107–48.655)	< 0.001
Intercept	−3.958	1.091	0.019	< 0.001

*Note:* Tg, thyroglobulin.

Abbreviations: CI, confidence interval; OR, odds ratio; SE, standard error.

The final multivariate model was expressed as follows:

(1) Logitp=−3.9580.4280.2400.5561.1592.509−×age/10+×log2Tg+×diameter/10+×hypoechogenicity+×irregularity,

where hypoechogenicity and irregularity were coded as 1 and their counterparts as 0. A nomogram was subsequently constructed to quantify the contribution of each predictor to FTC risk (Figure 3).

**FIGURE 3:**
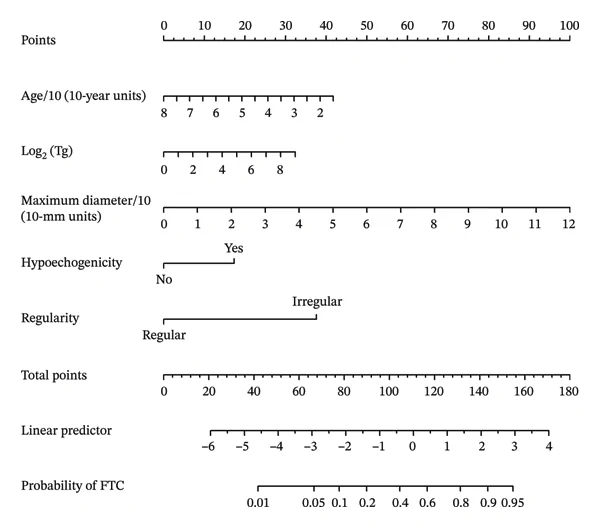
The nomogram for predicting follicular thyroid carcinoma based on clinical and ultrasonographic features. The initial number line shows a score range (0–100) for the variable. Subsequent lines (2–6) depict the 5 independent risk factors, each associated with a score located on the first line. The cumulative scores of all factors constitute the total score for each nodule, and the total score can be found on the 7th line. The 8th line represents the log‐odds derived from the logistic regression model, serving as an intermediate transformation step between total points and predicted probability. The 9th line represents the probability of follicular thyroid carcinoma diagnosis. FTC, follicular thyroid carcinoma; Tg, thyroglobulin.

Sensitivity analysis using Firth penalized logistic regression yielded effect estimates that were similar in the direction to those from the conventional model (Supporting Table S1). For irregularity, the conventional estimate was OR 12.295 (95% CI: 3.107–48.655), whereas the Firth penalized estimate was OR 10.679 (95% CI: 3.022–43.716; *p* < 0.001). Estimates for age, log_2_ (Tg), maximum diameter, and hypoechogenicity were also materially similar after penalization. Thus, bias‐reduced estimation produced modest attenuation of the irregularity effect but did not alter the direction of the association; nevertheless, the wide CI indicates that the magnitude of this effect remains imprecisely estimated.

### 3.4. Evaluation and Validation of the Diagnostic Model

ROC analysis demonstrated good discriminative ability of the model, with an AUC of 0.822 (95% CI: 0.753–0.891) in the training cohort (Figure 4a) and 0.801 (95% CI: 0.686–0.917) in the temporal validation cohort (Figure 4b). Calibration analysis on the temporal validation cohort yielded a slope of 0.926 (95% CI: 0.491–1.813) and an intercept of 0.369 (95% CI: −0.427–1.464). Point estimates approximated the ideal values (slope = 1, intercept = 0), and both CIs included the reference. However, the wide intervals indicate limited precision of the calibration estimates, warranting confirmation in larger external cohorts. The Brier score was 0.177 (Figure 5a,b). DCA showed differences in estimated net benefit between the combined model and the retrospectively reconstructed ACR TI‐RADS comparator across the evaluated threshold range, with the combined model showing higher estimated net benefit at several thresholds (Figure 6a,b). These exploratory findings should be interpreted as supportive benchmarking rather than evidence of superiority. The optimal cut‐off value of 0.2628 was derived exclusively from the training cohort by maximizing the Youden index and was subsequently applied without modification to the temporal validation cohort. At this operating point, the temporal validation cohort showed a sensitivity of 71.43% (95% CI: 50.00%–86.20%) and a specificity of 75.61% (95% CI: 60.70%–86.20%). The observed PPV and NPV were 60.00% and 83.78%, respectively, with a PLR of 2.929 and an NLR of 0.378 (Table 5, Panel A). Importantly, among patients classified as low risk at this threshold, 6 of 37 (16.22%) nevertheless had FTC, indicating that this operating point is insufficient for use as a rule‐out threshold. Because predictive values are prevalence‐dependent, PPV and NPV were additionally standardized across pretest probabilities ranging from 10% to 30% (Table 5, Panel B). At pretest probabilities of 10%, 20%, and 30%, the corresponding standardized PPVs were 24.55%, 42.27%, and 55.66%, whereas the standardized NPVs were 95.97%, 91.37%, and 86.06%, respectively. An exploratory rule‐out‐oriented threshold of 0.1229 was derived in the training cohort as the highest threshold achieving a sensitivity of at least 90%. At this threshold, sensitivity and specificity in the training cohort were 91.67% and 48.59%, respectively. When the threshold was applied unchanged to the temporal validation cohort, sensitivity was 90.48% and specificity was 48.78%, with an observed NPV of 90.91% (Table 5, Panel C). Thus, the gain in sensitivity was accompanied by a substantial reduction in specificity. However, because the study excluded nonoperated indeterminate nodules, selection and spectrum effects may limit the transportability of these estimates, precluding any direct inference regarding the model’s ability to reduce diagnostic surgery rates in unselected clinical populations.

**FIGURE 4:**
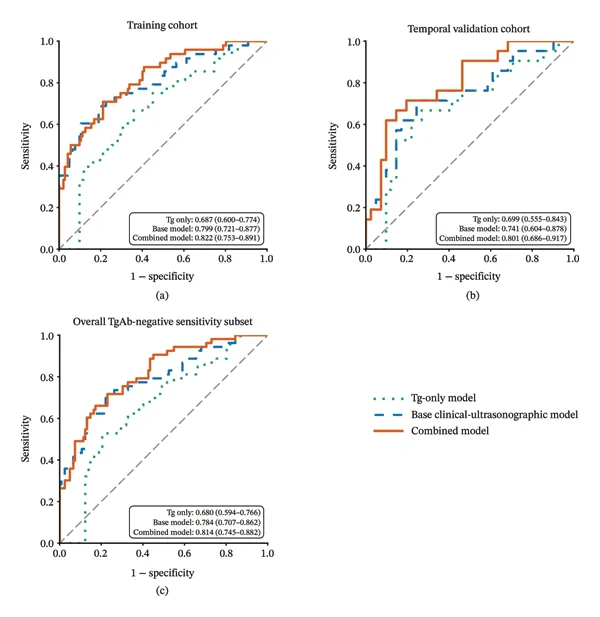
ROC curves of the nomogram for predicting follicular thyroid carcinoma. (a) ROC curve in the training cohort, (b) ROC curve in the temporal validation cohort, and (c) ROC curve in the serum TgAb‐negative subgroup. ROC, receiver operating characteristic; TgAb, thyroglobulin antibody.

**FIGURE 5:**
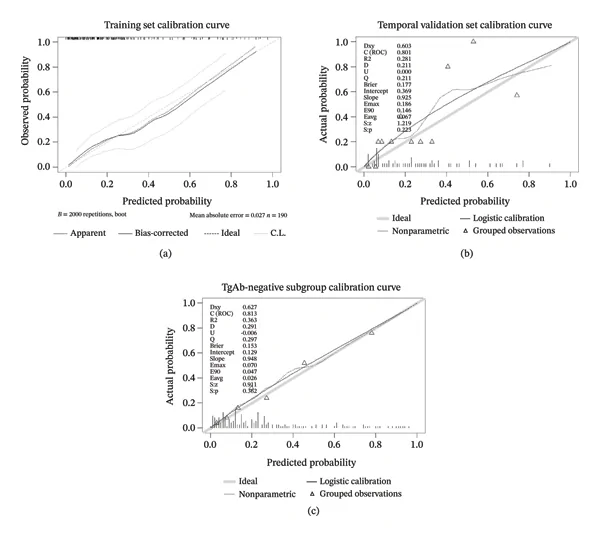
Calibration curves of the nomogram for predicting follicular thyroid carcinoma. (a) Calibration curve in the training cohort, (b) calibration curve in the temporal validation cohort, and (c) calibration curve in the serum TgAb‐negative subgroup. The *x*‐axis represents the overall predicted probability of follicular thyroid carcinoma, and the *y*‐axis represents the actual probability. The model calibration is indicated by the degree of fit of the curve and the diagonal line. FTC, follicular thyroid carcinoma; TgAb, thyroglobulin antibody.

**FIGURE 6:**
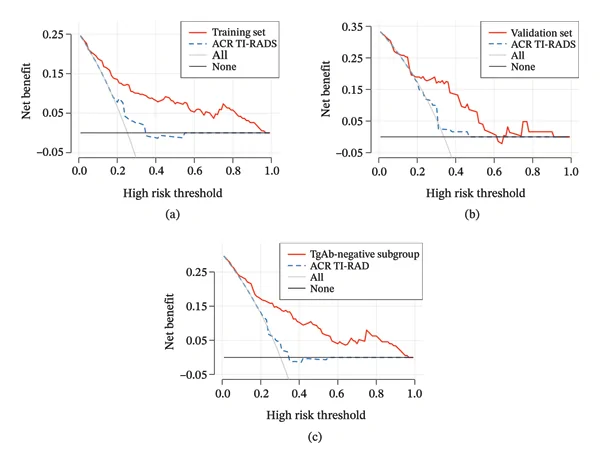
Decision curve analysis comparing the combined model with the retrospectively reconstructed ACR TI‐RADS comparator for predicting follicular thyroid carcinoma. (a) Training cohort, (b) temporal validation cohort, and (c) overall TgAb‐negative sensitivity subset. The combined model and ACR TI‐RADS comparator were fitted in the training cohort, and the fitted coefficients were applied without modification to the temporal validation cohort. “All” represents the strategy in which all patients are considered to have FTC, whereas “None” represents the strategy in which no patients are considered to have FTC. FTC, follicular thyroid carcinoma; DCA, decision curve analysis; ACR TI‐RADS, American College of Radiology Thyroid Imaging Reporting and Data System; TgAb, thyroglobulin antibody.

**TABLE 5:** Operating point performance and Bayes‐derived predictive values of the combined prediction model.

**Cohort**	**AUC (95% CI)**	**Sensitivity (95% CI)**	**Specificity (95% CI)**	**PPV (95% CI)**	**NPV (95% CI)**	**PLR (95% CI)**	**NLR (95% CI)**

*Panel A. Observed cohort-specific performance at the training-derived Youden threshold (0.2628)*							
Temporal validation cohort	0.801 (0.686–0.917)	0.714 (0.500–0.862)	0.756 (0.607–0.862)	0.600 (0.407–0.766)	0.838 (0.689–0.924)	2.929 (1.602–5.352)	0.378 (0.188–0.760)
Overall TgAb‐negative sensitivity subset	0.814 (0.745–0.882)	0.717 (0.584–0.820)	0.771 (0.688–0.836)	0.576 (0.456–0.688)	0.862 (0.785–0.915)	3.124 (2.165–4.507)	0.367 (0.237–0.570)
**Pretest probability**	**Standardized PPV (95% CI)**			**Standardized NPV (95% CI)**			

*Panel B. Bayes-derived PPV and NPV across specified pretest probabilities at the fixed 0.2628 threshold in the temporal validation cohort*							
10%	0.246 (0.158–0.394)			0.960 (0.933–0.986)			
15%	0.341 (0.230–0.508)			0.937 (0.897–0.978)			
20%	0.423 (0.297–0.594)			0.914 (0.860–0.969)			
25%	0.494 (0.361–0.661)			0.888 (0.822–0.960)			
30%	0.557 (0.420–0.715)			0.861 (0.782–0.949)			

**Cohort**	**Threshold**	**Sensitivity (95% CI)**	**Specificity (95% CI)**	**PPV (95% CI)**	**NPV (95% CI)**	**PLR (95% CI)**	**NLR (95% CI)**

*Panel C. Post hoc rule-out-oriented operating point derived in the training cohort and applied unchanged to temporal validation*							
Training cohort	0.1229	0.917 (0.804–0.967)	0.486 (0.405–0.567)	0.376 (0.294–0.466)	0.945 (0.867–0.978)	1.783 (1.488–2.137)	0.171 (0.066–0.445)
Temporal validation cohort	0.1229	0.905 (0.711–0.973)	0.488 (0.343–0.635)	0.475 (0.329–0.625)	0.909 (0.722–0.975)	1.766 (1.271–2.456)	0.195 (0.050–0.757)

*Note:* In Panel B, PPV and NPV were estimated by Bayes’ theorem using the sensitivity and specificity observed in the temporal validation cohort at the fixed threshold of 0.2628; 95% CIs were obtained from 2000 stratified bootstrap resamples. In Panel C, the rule‐out‐oriented threshold was derived exclusively in the training cohort as the highest threshold achieving sensitivity ≥ 90% and was applied unchanged to the temporal validation cohort. TgAb, thyroglobulin antibody.

Abbreviations: AUC, area under the curve; CI, confidence interval; FA, follicular adenoma; FTC, follicular thyroid carcinoma; NLR, negative likelihood ratio; NPV, negative predictive value; PLR, positive likelihood ratio; PPV, positive predictive value.

### 3.5. Sensitivity Analyses for Tg Values at the Upper Reporting Limit

To address the concern that upper‐limit censoring at 500 ng/mL might bias effect estimates, sensitivity analyses were performed to assess the robustness of the log_2_ (Tg) association in the primary multivariable model. In the unadjusted analysis, patients with FTC were significantly more likely than those with FA to present with Tg values at the censoring threshold (30.43% vs 9.84%; OR = 3.984, 95% CI: 1.856–8.652; Fisher’s exact *p* < 0.001; Table 2). This unadjusted association between markedly elevated Tg and FTC provided additional descriptive support for evaluating log_2_ (Tg) as a candidate predictor in the multivariable model.

To evaluate whether the observed association between log_2_ (Tg) and FTC was driven by observations at the upper reporting limit, a binary indicator variable (1 if Tg = 500 ng/mL; 0 otherwise) was incorporated into the primary model. In this adjusted model, the effect of log_2_ (Tg) remained directionally positive (OR per doubling = 1.232; 95% CI: 0.946–1.605; *p* = 0.121), whereas the indicator variable showed no independent association with FTC diagnosis (OR = 1.281, 95% CI: 0.380–4.321; *p* = 0.689). The addition of the indicator did not significantly improve model fit, as assessed by the likelihood ratio test (*p* = 0.690). The attenuation of statistical significance for log_2_ (Tg) in this model may partly reflect collinearity between the continuous Tg values and the censoring indicator, rather than an absence of a true effect. Model discrimination remained strong in both the training set (AUC = 0.826, 95% CI: 0.757–0.894) and the validation set (AUC = 0.794, 95% CI: 0.676–0.913). These findings did not provide clear evidence that adding an upper‐limit censoring indicator materially improved model fit. Model discrimination remained broadly similar to that of the primary model, supporting the adequacy of the continuous log_2_‐transformed specification.

In a further sensitivity analysis, all 39 patients with Tg values recorded at the upper reporting limit (500 ng/mL) were excluded, and the prespecified model was refitted. After exclusion, 161 patients with 33 FTC events remained in the training cohort, and 52 patients with 15 FTC events remained in the temporal validation cohort. The Tg effect estimate again remained positive but did not reach statistical significance (OR per doubling: 1.224, 95% CI: 0.925–1.619; *p* = 0.157). The corresponding AUCs were 0.823 (95% CI: 0.738–0.907) in the reduced training cohort and 0.831 (95% CI: 0.692–0.969) in the reduced temporal validation cohort. The Tg estimate remained positive, and model discrimination was broadly preserved, but the association did not reach statistical significance after exclusion of values recorded at the upper reporting limit. The direction of the Tg estimate and the broadly preserved model discrimination suggest that the observed Tg signal was not solely driven by values recorded at the upper reporting limit. However, the loss of statistical significance after exclusion of these observations indicates uncertainty regarding the strength and independence of the Tg association.

Taken together, these sensitivity analyses suggest that upper‐limit censoring alone is unlikely to account for the observed Tg signal. The Tg effect remained directionally positive across analyses, and overall model discrimination was broadly preserved. However, the Tg association was no longer statistically significant after either inclusion of an upper‐limit indicator (*p* = 0.121) or exclusion of observations recorded at 500 ng/mL (*p* = 0.157). These findings therefore support cautious retention of log_2_‐transformed Tg in the exploratory prediction model while indicating uncertainty regarding the strength and independence of its association with FTC (Table 2).

### 3.6. Subgroup Analysis in TgAb‐Negative Patients

Given the potential analytical interference of TgAb on serum Tg measurements, a subgroup analysis, not an independent validation, was conducted in TgAb‐negative patients (TgAb ≤ 115.00 IU/mL), including 53 FTC and 122 FA cases treated between January 2016 and August 2024.

### 3.7. Clinical and Ultrasonographic Characteristics

Baseline demographic characteristics, thyroid serological parameters, and ultrasonographic features of the TgAb‐negative subgroup are summarized in Table 6. Consistent with findings from the training cohort, no significant difference in sex distribution was observed between FTC and FA patients. Younger age and higher serum Tg levels were significantly associated with FTC, whereas no significant differences were observed in preoperative FT3, FT4, TSH, TgAb, or TPOAb levels. Compared with FA, FTC was more likely to present with larger nodule diameter (*p* < 0.001), hypoechogenicity (*p* = 0.009), calcification (*p* = 0.009), irregularity (*p* = 0.004), and rich blood supply (*p* = 0.034). No statistically significant differences were observed in nodule number, location, composition, margin, taller than wide, or peripheral halo (*p* > 0.05).

**TABLE 6:** Clinical and ultrasonographic characteristics of patients in the serum TgAb‐negative subgroup.

	FTC (*n* = 53)	FA (*n* = 122)	*p*
Gender, *n* (%)			0.162
Male	18 (34.00%)	29 (23.80%)	
Female	35 (66.00%)	93 (76.20%)	
Age (years)	41.09 ± 16.71	47.32 ± 14.50	0.014^∗^
FT3 (pmol/L)	4.82 (4.30–5.25)	4.89 (4.49–5.29)	0.381
FT4 (pmol/L)	15.80 (14.18–17.82)	15.97 (14.80–17.57)	0.539
TSH (mIU/L)	1.79 (1.16–2.83)	1.79 (1.22–2.76)	0.903
Tg (ng/mL)	234.70 (72.20–500.00)	73.53 (26.01–206.80)	< 0.001^∗^
TgAb (IU/mL)	16.50 (13.35–19.30)	15.60 (12.00–19.48)	0.466
TPOAb (IU/mL)	10.00 (7.20–14.70)	10.20 (8.08–16.95)	0.196
Maximum diameter (mm)	47.00 (39.25–56.90)	39.00 (28.73–47.00)	< 0.001^∗^
Number, *n* (%)			0.298
Single	18 (34.00%)	32 (26.20%)	
Multiple	35 (66.00%)	90 (73.80%)	
Location, *n* (%)			0.616
Left	23 (43.40%)	48 (39.30%)	
Right	30 (56.60%)	74 (60.70%)	
Isthmus	0	0	
Composition, *n* (%)			0.350
Solid	31 (58.50%)	62 (50.80%)	
Cystic or mixed	22 (41.50%)	60 (49.20%)	
Echogenicity, *n* (%)			
Type 1			0.028^∗^
Isoechogenicity	19 (35.80%)	64 (52.50%)	
Hypoechogenicity	34 (64.20%)	52 (42.60%)	
Hyperechogenicity	0	5 (4.10%)	
Mixed echogenicity	0	1 (0.80%)	
Type 2			
Hypoechogenicity, n (%)			0.009^∗^
Present	34 (64.20%)	52 (42.60%)	
Absent	19 (35.80%)	70 (57.40%)	
Calcification, *n* (%)			
Type 1			0.030^∗^
Absent	26 (49.10%)	85 (69.70%)	
Microcalcification	12 (22.60%)	21 (17.20%)	
Macrocalcification	14 (26.40%)	15 (12.30%)	
Peripheral calcification	1 (1.90%)	1 (0.80%)	
Type 2			0.009^∗^
Present	27 (50.90%)	37 (30.30%)	
Absent	26 (49.10%)	85 (69.70%)	
Regularity, *n* (%)			0.004^∗^
Regular	43 (81.10%)	117 (95.90%)	
Irregular	10 (18.90%)	5 (4.10%)	
Margin, *n* (%)			0.304
Smooth	46 (86.80%)	112 (91.80%)	
Irregular	7 (13.20%)	10 (8.20%)	
Taller than wide, *n* (%)			0.774
Present	1 (1.90%)	5 (4.10%)	
Absent	52 (98.10%)	117 (95.90%)	
Peripheral halo, *n* (%)			0.964
Present	12 (22.60%)	28 (23.00%)	
Absent	41 (77.40%)	94 (77.00%)	
Blood supply, *n* (%)			0.034^∗^
Rich	49 (92.50%)	97 (79.50%)	
Scarce	4 (7.50%)	25 (20.50%)	

*Note:* FT3, free triiodothyronine; FT4, free thyroxine; Tg, thyroglobulin; TgAb, thyroglobulin antibody; TPOAb, thyroid peroxidase antibody.

Abbreviations: FA, follicular adenoma; FTC, follicular thyroid carcinoma; TSH, thyroid‐stimulating hormone.

^∗^Statistically significant difference.

### 3.8. Model Performance in the TgAb‐Negative Subgroup

The model demonstrated good discrimination, with an AUC of 0.814 (95% CI: 0.745–0.882) (Figure 4c). Calibration analysis yielded a slope of 0.948 (95% CI: 0.702–1.349) and an intercept of 0.129 (95% CI: −0.306–0.686). Point estimates were close to the ideal values, and both CIs included the reference, suggesting no evidence of overfitting or systematic bias. However, the intervals were moderately wide, indicating limited precision and warranting confirmation in larger external cohorts. The Brier score was 0.153 (Figure 5c). In the TgAb‐negative sensitivity subset, DCA showed a similar threshold‐dependent pattern of estimated net benefit between the combined model and the reconstructed ACR TI‐RADS comparator; this exploratory comparison should likewise be interpreted as supportive benchmarking (Figure 6c). A sensitivity analysis was performed in the TgAb‐negative subgroup using the same cut‐off of 0.2628. Compared with the temporal validation cohort, the combined model showed comparable performance, with a sensitivity of 71.70% (95% CI: 58.40%–82.00%), specificity of 77.05% (95% CI: 68.80%–83.60%), PPV of 57.58% (95% CI: 45.60%–68.80%), and NPV of 86.24% (95% CI: 78.50%–91.50%). The PLR and NLR were 3.124 (95% CI: 2.165–4.507) and 0.367 (95% CI: 0.237–0.570), respectively (Table 5, Panel A).

### 3.9. Comparative Model Performance

#### 3.9.1. Head‐to‐Head Model Comparisons

Head‐to‐head comparisons were performed among four prespecified models: (1) a Tg‐only model (log_2_‐transformed serum Tg), (2) a base clinical‐ultrasonographic model (age, maximum nodule diameter, hypoechogenicity, and irregularity), (3) a combined model incorporating all base model predictors plus log_2_‐transformed Tg, and (4) the retrospectively reconstructed ACR TI‐RADS comparator (composition, echogenicity, taller than wide, margin, echogenic foci). The Tg‐only, base, and combined models were fitted in the training cohort as described above, and the ACR TI‐RADS comparator was additionally constructed for benchmarking against an established ultrasonographic risk stratification system. All model coefficients were applied without modification to the temporal validation cohort.

#### 3.9.2. Performance in the Training Cohort

In the training cohort, the combined model had a numerically higher AUC than the base model (0.822 vs 0.799), but the difference was not statistically significant (ΔAUC: 0.023, 95% CI: –0.007–0.053; DeLong *p* = 0.139). The IDI was also not statistically significant (0.017, 95% CI: –0.006–0.040), whereas the continuous NRI was positive (0.433, 95% CI: 0.113–0.740), indicating inconsistency across incremental performance metrics. Compared with the Tg‐only model, the combined model showed a higher AUC (0.822 vs 0.687; ΔAUC: 0.135, 95% CI: 0.043–0.227; *p* = 0.004), with positive NRI and IDI. Compared with the reconstructed ACR TI‐RADS comparator, the combined model also showed a higher AUC (0.822 vs 0.633; ΔAUC: 0.189, 95% CI: 0.096–0.283; *p* < 0.001), with a continuous NRI of 0.896 (95% CI: 0.602–1.187) and an IDI of 0.284 (95% CI: 0.198–0.370) (Table 7, Panel B).

**TABLE 7:** Discrimination and comparative predictive performance of the Tg‐only, base clinical‐ultrasonographic, ACR TI‐RADS, and combined models in the training cohort, temporal validation cohort, and TgAb‐negative sensitivity subset.

**Model**	**Predictors**	**Training AUC (95% CI)**	**Temporal validation AUC (95% CI)**		**Overall TgAb-negative subset AUC (95% CI)**	

*Panel A. Discrimination of the four models*						
Tg‐only model	log_2_‐transformed Tg	0.687 (0.600–0.774)	0.699 (0.555–0.843)		0.680 (0.594–0.766)	
Base clinical‐ultrasonographic model	Age per 10‐year increase; maximum diameter per 10‐mm increase; hypoechogenicity; irregular shape	0.799 (0.721–0.877)	0.741 (0.604–0.878)		0.784 (0.707–0.862)	
ACR TI‐RADS comparator	Composition; echogenicity; taller than wide; margin; echogenic foci	0.633 (0.545–0.721)	0.692 (0.547–0.836)		0.624 (0.536–0.712)	
Combined model	Base model plus log_2_‐transformed Tg	0.822 (0.753–0.891)	0.801 (0.686–0.917)		0.814 (0.745–0.882)	

**Comparison**	**Reference AUC**	**Combined-model AUC**	**Delta AUC (95% CI)**	**DeLong** **p** **value**	**Continuous NRI (95% CI)**	**IDI (95% CI)**

*Panel B. Incremental performance in the training cohort (apparent performance)*						
Combined vs base model	0.799	0.822	0.023 (−0.007–0.053)	0.139	0.433 (0.113–0.740)	0.017 (−0.006–0.040)
Combined vs Tg‐only model	0.687	0.822	0.135 (0.043–0.227)	0.004	0.743 (0.424–1.024)	0.230 (0.149–0.309)
Combined vs ACR TI‐RADS	0.633	0.822	0.189 (0.096–0.283)	< 0.001	0.896 (0.602–1.187)	0.284 (0.198–0.370)

**Comparison**	**Reference AUC**	**Combined-model AUC**	**Delta AUC (95% CI)**	**DeLong** **p** **value**	**Continuous NRI (95% CI)**	**IDI (95% CI)**

*Panel C. Incremental performance in the temporal validation cohort*						
Combined vs base model	0.741	0.801	0.060 (0.010–0.111)	0.020	0.506 (−0.021–1.034)	0.026 (−0.021–0.072)
Combined vs Tg‐only model	0.699	0.801	0.102 (−0.065–0.269)	0.230	0.702 (0.221–1.182)	0.147 (0.032–0.268)
Combined vs ACR TI‐RADS	0.692	0.801	0.110 (−0.040–0.260)	0.152	0.602 (0.116–1.127)	0.188 (0.082–0.296)

**Comparison**	**Reference AUC**	**Combined-model AUC**	**Delta AUC (95% CI)**	**DeLong** **p** **value**	**Continuous NRI (95% CI)**	**IDI (95% CI)**

*Panel D. Incremental performance in the overall TgAb-negative sensitivity subset (2016–2024)*						
Combined vs base model	0.784	0.814	0.030 (−0.001–0.060)	0.055	0.376 (0.068–0.673)	0.017 (−0.006–0.039)
Combined vs Tg‐only model	0.680	0.814	0.134 (0.042–0.225)	0.004	0.775 (0.466–1.052)	0.211 (0.129–0.289)
Combined vs ACR TI‐RADS	0.624	0.814	0.189 (0.093–0.286)	< 0.001	0.903 (0.619–1.174)	0.267 (0.190–0.347)

*Note:* ACR TI‐RADS scores were retrospectively reconstructed from the recorded ultrasonographic descriptors. The ACR TI‐RADS comparator was fitted in the training cohort and applied without modification to the temporal validation cohort. Continuous NRI and IDI 95% CIs were derived from 2000 stratified bootstrap resamples. Comparisons with ACR TI‐RADS should be interpreted as supportive benchmarking. Tg, thyroglobulin; TgAb, thyroglobulin antibody.

Abbreviations: AUC, area under the curve; CI, confidence interval; FA, follicular adenoma; FTC, follicular thyroid carcinoma; IDI, integrated discrimination improvement; NRI, net reclassification improvement.

#### 3.9.3. Performance in the Temporal Validation Cohort

In the temporal validation cohort, the combined model had a higher AUC than the base model (0.801 vs 0.741; ΔAUC: 0.060, 95% CI: 0.010–0.111; *p* = 0.020), although the continuous NRI (0.506, 95% CI: –0.021–1.034) and IDI (0.026, 95% CI: –0.021–0.072) were imprecisely estimated and included the null. Compared with the Tg‐only model, the AUC difference was not statistically significant (0.801 vs 0.699; ΔAUC: 0.102, 95% CI: –0.065–0.269; *p* = 0.230), whereas the continuous NRI and IDI were positive. Compared with the reconstructed ACR TI‐RADS comparator, the combined model had a higher observed AUC in the temporal validation cohort (0.801 vs 0.692; ΔAUC: 0.110, 95% CI: –0.040–0.260), but the DeLong comparison was not statistically significant (*p* = 0.152). The continuous NRI was 0.602 (95% CI: 0.116–1.127), and the IDI was 0.188 (95% CI: 0.082–0.296). Thus, although reclassification and integrated discrimination metrics favored the combined model, superiority in AUC over ACR TI‐RADS was not established in the temporal validation cohort (Table 7, Panel C).

#### 3.9.4. Performance in the Overall TgAb‐Negative Sensitivity Subset

In the overall TgAb‐negative sensitivity subset, the AUC difference between the combined and base models was small and did not reach statistical significance (0.814 vs 0.784; ΔAUC: 0.030, 95% CI: –0.001–0.060; *p* = 0.055), although the continuous NRI was positive. Compared with the Tg‐only model, the combined model showed a higher AUC (0.814 vs 0.680; ΔAUC: 0.134, 95% CI: 0.042–0.225; *p* = 0.004). Compared with the reconstructed ACR TI‐RADS comparator, the combined model showed a higher AUC (0.814 vs 0.624; ΔAUC: 0.189, 95% CI: 0.093–0.286; *p* < 0.001), with a continuous NRI of 0.903 (95% CI: 0.619–1.174) and an IDI of 0.267 (95% CI: 0.190–0.347) (Table 7, Panel D). Because this subset comprised patients from the full study period, these results represent a sensitivity analysis rather than independent validation. In addition, comparisons with retrospectively reconstructed ACR TI‐RADS should be regarded as supportive benchmarking rather than evidence of superiority over prospectively assigned ACR TI‐RADS.

## 4. Discussion

Preoperative differentiation of FNs into benign and malignant entities remains a well‐recognized clinical challenge. When the malignant potential of a thyroid nodule cannot be reliably determined, diagnostic surgical resection is often recommended. This strategy may result in overtreatment, exposing patients to unnecessary surgical risks, increased healthcare costs, and potential long‐term consequences, including recurrent laryngeal nerve injury and lifelong thyroid hormone replacement with ongoing biochemical monitoring. In this context, evaluating noninvasive models for preoperative risk stratification is clinically relevant. In the present study, we evaluated whether routinely available clinical and ultrasonographic variables could stratify the risk of FTC among patients with follicular thyroid neoplasms who ultimately underwent surgical resection. In the training cohort, age (per 10‐year increase), elevated log_2_‐transformed serum Tg, larger maximum diameter (per 10‐mm increase), hypoechogenicity, and irregularity were identified as independent predictors of FTC. The model demonstrated good discriminative performance, with an AUC of 0.822 (95% CI: 0.753–0.891) in the training cohort and 0.801 (95% CI: 0.686–0.917) in the temporal validation cohort.

The association between age and FTC remains controversial. In the present study, younger age was identified as an independent risk factor for FTC, consistent with the findings of Lee et al. [26]. The median age of 39 years among FTC patients in the training cohort aligns closely with the observations of Van et al. [27], who suggested that an age threshold of approximately 40 years may be more appropriate for staging and prognostic stratification in FTC. In contrast, Xu et al. [28] reported no significant age difference between FTC and FA, suggesting that age alone may not be a universal predictor. These discrepancies may be attributable to differences in study design, patient populations, and sample sizes, underscoring the need for further large‐scale investigations to clarify the role of age in FTC risk stratification.

Thyroglobulin (Tg) is a thyroid tissue‐specific protein and is widely used as a biomarker for detecting residual or recurrent DTC after thyroidectomy and radioactive iodine therapy [29]. In the present study, higher preoperative serum Tg was associated with FTC after adjustment for age, maximum nodule diameter, hypoechogenicity, and irregularity. When modeled as a log_2_‐transformed continuous variable, each doubling of Tg was associated with a 27.20% increase in the odds of FTC. Within the nested modeling framework, addition of Tg was associated with an increase in AUC from 0.741 to 0.801 in the temporal validation cohort. When benchmarked separately against the retrospectively reconstructed ACR TI‐RADS comparator, the combined model also showed a higher AUC (0.801 vs 0.692), although the AUC difference did not reach statistical significance (*p* = 0.152). Continuous NRI and IDI favored the combined model, but these measures, particularly continuous NRI, should be interpreted cautiously and together with the nonsignificant DeLong comparison. Accordingly, the present findings suggest possible incremental risk stratification information beyond reconstructed ACR TI‐RADS but do not establish superiority over routine ACR TI‐RADS assessment. Nevertheless, the observed association should not be interpreted as definitive evidence that Tg is independent of all effects related to tumor size or other unmeasured biological characteristics.

The Tg findings also require cautious interpretation because 30.40% of FTC cases and 9.80% of FA cases had Tg values recorded at the upper reporting limit of 500 ng/mL, preventing differentiation among very high concentrations. In sensitivity analyses, the Tg effect estimates remained positive and were similar in magnitude to the primary estimate, but their CIs included unity after either adding an upper‐limit indicator or excluding observations recorded at 500 ng/mL. Model discrimination remained broadly similar across these analyses, suggesting that overall predictive performance was not substantially altered, although the statistical evidence for an independent Tg association was attenuated. This uncertainty is also reflected by the moderate bootstrap inclusion frequency of 54.50% and the nonsignificant overall association in the multivariable RCS analysis (*p* overall = 0.096). In addition, quantitative Tg measurements are assay dependent, and the present findings should therefore be interpreted in the context of the analytical platform and measuring range used in our laboratory. Potential biological explanations remain exploratory. Li et al. [30] reported an association between serum Tg and hypoxia‐inducible factor‐1*α*, while increased HIF‐1*α* expression has been associated with vascular invasion and distant metastasis in FTC [31]. Tumor hypoxia, follicular cell proliferation, capsular or vascular invasion, and disruption of follicular architecture could plausibly increase the release of Tg into the circulation; however, these mechanisms were not directly evaluated in the present study and require further investigation.

A sensitivity analysis performed in the TgAb‐negative subgroup, applying the same cut‐off value of 0.2628, yielded performance metrics broadly comparable to those of the temporal validation cohort. Because this subset was drawn from the overall study cohort, it should not be interpreted as independent validation or evidence of generalizability. External validation in independent multicenter cohorts remains necessary. While these findings are encouraging, our conclusions remain tempered: the consistent performance observed in this subgroup demonstrates the model’s robustness in TgAb‐negative samples within the current cohort, but does not constitute definitive evidence of external generalizability.

Tumor size is a well‐established parameter in thyroid cancer staging systems and is generally associated with tumor aggressiveness [32]. In the present study, a larger maximum diameter (per 10‐mm increase) was identified as an independent predictor of FTC, consistent with a previous report [33]. Follicular thyroid neoplasms larger than 4 cm have been shown to carry a higher risk of malignancy and are often recommended for surgical management. These findings further support the inclusion of tumor diameter as a key component of preoperative risk assessment.

Ultrasonographic echogenicity is an important indicator of thyroid nodule malignancy, with hypoechogenicity traditionally considered a suspicious feature [34]. In our cohort, hypoechogenicity was significantly more frequent in FTC (68.75%) than in FA (47.90%) and was associated with substantially 3.188‐fold higher odds of malignancy, corroborating previous studies [9, 35]. This finding reinforces the diagnostic value of echogenicity assessment in FNs, despite its limited specificity when used in isolation.

Calcification has also been regarded as a suspicious ultrasonographic feature of thyroid malignancy. Consistent with previous reports [28, 36], calcification showed statistical significance in univariate analysis in the training cohort; however, it was excluded from the final model, as its inclusion frequency (34.60%) fell below the prespecified stability threshold. Calcification may be more prevalent in FTC due to rapid tumor growth leading to ischemic necrosis and dystrophic calcification. Nevertheless, these findings suggest that calcification alone lacks sufficient discriminatory power and should be interpreted in conjunction with other ultrasonographic features and clinical parameters.

Morphological irregularity is a recognized suspicious feature of thyroid nodules [35, 37]. In the present study, irregularity was associated with FTC in the conventional multivariable model; however, it was uncommon in the training cohort (11 of 48 FTC cases and 7 of 142 FA cases), and the corresponding estimate was large and imprecise (OR: 12.295, 95% CI: 3.107–48.655), raising concern for small‐sample and sparse‐data inflation. In the Firth penalized sensitivity analysis, the estimate was modestly attenuated to OR 10.679 (95% CI: 3.022–43.716), while remaining statistically supported, suggesting that the direction of the association was robust to bias‐reduced estimation but that its magnitude should be interpreted cautiously. Because assessment of shape may also be subjective, we additionally evaluated inter‐reader reproducibility in an independently rereviewed subset. Agreement for irregularity was high (observed agreement 96.70%; Cohen’s *κ* 0.880, 95% CI: 0.765–0.995). Nevertheless, the limited number of irregular cases, wide CIs, and use of archived static images should all be considered when interpreting the predictive contribution of this feature.

Several limitations should be acknowledged. First, the study is subject to spectrum and selection bias because only surgically resected, histologically confirmed nodules were included. This enriched the cohort for relatively large or clinically concerning lesions and limits the representativeness of consecutive patients with cytologically indeterminate nodules in whom the decision to operate remains uncertain. The temporal validation cohort was derived from the same institution and was subject to the same selection process and therefore does not establish broader generalizability. Accordingly, the model should be interpreted as an exploratory risk stratification tool for surgically selected patients rather than as a validated tool for guiding diagnostic surgery in unselected populations. Second, the ultrasonographic features were retrospectively derived from archived static images and written reports, which may not fully capture information available during real‐time dynamic scanning. Although inter‐reader agreement was high, prospective studies using standardized image acquisition and prospectively assigned ACR TI‐RADS assessments are needed to confirm reproducibility and clinical applicability. Accordingly, the ACR TI‐RADS comparison should be regarded as supportive benchmarking rather than evidence of superiority over prospectively assigned ACR TI‐RADS. Third, the limited number of FTC events relative to the predictors considered may increase the risk of small‐sample bias, overfitting, and coefficient instability, particularly for sparsely distributed features such as irregularity. Although Firth penalized regression yielded similar estimates, confirmation in larger independent cohorts is warranted. Fourth, although internal and temporal validation were performed, external validation using independent datasets from other institutions remains necessary. Future multicenter studies incorporating standardized ultrasound acquisition protocols and advanced imaging techniques may further enhance the robustness and clinical applicability of the proposed model. Fifth, serum Tg values above 500 ng/mL were recorded at the assay’s upper reporting limit, preventing differentiation among very high concentrations. Although sensitivity analyses were performed, residual bias related to upper‐limit censoring cannot be excluded. Furthermore, differences in assay calibration and analytical platforms may limit the direct transportability of absolute Tg values across laboratories. Accordingly, the model should not be used to rule out FTC, defer diagnostic surgery, or estimate absolute malignancy risk in unselected indeterminate nodules until it has been prospectively validated in consecutive multicenter cohorts that include both operated and nonoperated patients.

## 5. Conclusion

In this surgically selected cohort of patients with histologically confirmed follicular thyroid neoplasms, a model incorporating clinical and ultrasonographic variables demonstrated potentially useful discrimination between FTC and FA. The model should currently be regarded as an exploratory risk stratification tool for patients with characteristics similar to those included in this study. Prospective external validation in consecutive, unselected patients with cytologically indeterminate nodules is required before the model can be used to inform decisions to perform or avoid diagnostic surgery.

## Author Contributions

Minghui Ren and Mengshu Lu contributed equally to this work and share first authorship. Minghui Ren, Mengshu Lu, and Dai Cui conceived the study and designed the experimental workflow. Minghui Ren, Mengshu Lu, and Yajie Li performed sample collection. Minghui Ren, Mengshu Lu, and Dai Cui conducted data analysis and prepared figures. Minghui Ren and Dai Cui drafted the manuscript, and all authors participated in data interpretation and critical revision. Dai Cui supervised the study.

## Funding

This work was supported by the Thyroid Research Program of Young and Middle‐aged Physicians from the China Health Promotion Foundation and Foundation of Ili and Jiangsu Joint Institute of Health (Grant No. yl2020ms02).

## Disclosure

All authors read and approved the final manuscript.

## Ethics Statement

This retrospective study was conducted in accordance with the Declaration of Helsinki and its subsequent amendments. The study protocol was approved by the Ethics Committee of the First Affiliated Hospital with Nanjing Medical University (Ethics No. 2024‐SR‐455). Owing to the retrospective nature of the study, the requirement for written informed consent was waived by the Ethics Committee.

## Consent

Please see the Ethics Statement.

## Conflicts of Interest

The authors declare no conflicts of interest.

## Supporting Information

Additional supporting information can be found online in the Supporting Information section.

## Supporting information


**Supporting Information** Supporting Table S1. Sensitivity analysis comparing conventional maximum‐likelihood logistic regression and Firth penalized logistic regression in the training cohort. The table presents odds ratios, 95% confidence intervals, and *p* values for the five predictors included in the primary model.

## Data Availability

The data supporting the findings of this study are available from the corresponding author on reasonable request.
