# Supplementary material for: Development and Validation of a Clinical‐Ultrasonographic Model for Differentiating Follicular Thyroid Carcinoma From Adenoma in a Surgically Selected Cohort
Source: Int J Endocrinol. 2026 Nov 14;2026:2627742. doi: 10.1155/ije/2627742 (PMC13768868; doi:10.1155/ije/2627742)
Supplement: Supplementary file 1 [file IJE-2026-2627742-s001.docx]

Supplementary Table S1. Sensitivity analysis comparing conventional maximum-likelihood and Firth penalized logistic regression in the training cohort.

| **Variable** | **Conventional logistic regression** **OR (95% CI)** | **P** | **Firth penalized logistic regression** **OR (95% CI)** | **P** |
| --- | --- | --- | --- | --- |
| Age (per 10-year increase) | 0.652 (0.495–0.857) | 0.002 | 0.666 (0.504–0.861) | 0.002 |
| log₂(Tg) (per doubling) | 1.271 (1.024–1.579) | 0.030 | 1.255 (1.022–1.561) | 0.030 |
| Maximum diameter (per 10-mm increase) | 1.744 (1.308–2.326) | <0.001 | 1.686 (1.296–2.265) | <0.001 |
| Hypoechogenicity (Yes vs No) | 3.188 (1.357–7.486) | 0.008 | 3.019 (1.341–7.130) | 0.007 |
| Irregularity (Irregular vs Regular) | 12.295 (3.107–48.655) | <0.001 | 10.679 (3.022–43.716) | <0.001 |

OR, odds ratio; CI, confidence interval; Tg, thyroglobulin; FTC, follicular thyroid carcinoma. The training cohort included 190 patients with 48 FTC events. The same five predictors as in the primary multivariable model were included in both analyses. Conventional logistic-regression estimates are reproduced from the primary analysis (Table 4). Firth estimates were obtained by penalized maximum likelihood with profile penalized-likelihood 95% CIs and corresponding two-sided P values.
